# New species of *Metachela* Coquillett (Diptera, Empididae) from the Atlantic Forest, Brazil and a key to the Neotropical species

**DOI:** 10.3897/zookeys.714.11503

**Published:** 2017-11-07

**Authors:** Josenir Teixeira Câmara, Jose Albertino Rafael

**Affiliations:** 1 Instituto Nacional de Pesquisas da Amazônia, INPA, Coordenação de Biodiversidade, Caixa Postal 2223, 69080–971 Manaus, Amazonas, Brazil

**Keywords:** Empidoidea, Hemerodromiinae, *Metachela danitakiyae* sp. n., *Metachela spinulosa* sp. n., Neotropical realm, taxonomy

## Abstract

Two new species of *Metachela* Coquillett from the Brazilian Atlantic Forest, *M. danitakiyae* **sp. n.** from Rio de Janeiro and Minas Gerais and *M. spinulosa* **sp. n.** from Minas Gerais, are described and illustrated. A key to the Neotropical species is provided.

## Introduction

*Metachela* Coquillett [type species *M. collusor* (Melander)] is part of the tribe Hemerodromiini, and contains 12 previously described species, plus two species described here (see Table 1) with representatives in Western Europe, and the Neotropical and Nearctic regions (Yang et al. 2007). However, specimens of the genus are also known from Australia (pers. obs.). Collin (1933) and Smith (1962) described some South American species of *Metachela*, but considered them to be atypical in terms of antennal characters, thoracic shape, and head setation. MacDonald (1989) revised the genus; however, he covered only the three North American species.

**Table 1.** Checklist of world species of *Metachela*, with known sex and geographical records.

Species	Known sex		Geographical records
	Male	Female	
*Metachela albipes* (Walker, 1849)	x		Canada and USA
*M. barueri* Smith, 1962	x	x	Brazil (São Paulo)
*M. breviradius* Smith, 1962		x	Brazil (Santa Catarina)
*M. circumdata* Collin, 1933	x	x	Argentina (Bariloche)
*M. collusor* (Melander, 1902)	x	x	Canada and USA
*M. convexa* MacDonald, 1989	x		USA (California)
*M. danitakiyae* sp. n.	x	x	Brazil (Rio de Janeiro, Minas Gerais)
*M. flavella* Collin, 1933	x		Chile (Casa Pangue)
*M. hexachaeta* Collin, 1933	x	x	Chile (Casa Pangue, Puerto Varas, Puerto Montt, Peulla)
*M. inornata* Collin, 1933	x		Argentina (Bariloche)
*M. instabilis* Collin, 1933	x		Chile (Puerto Varas)
*M. nigriventris* (Loew, 1864)	x	x	Austria, Germany, Hungary, Italy?
*M. patula* Collin, 1933	x	x	Argentina (Bariloche)
*M. spinulosa* sp. n.	x	x	Brazil (Minas Gerais)

Yang et al. (2007) catalogued only eight species of *Metachela* for the Neotropical Realm; however, more species still await description as Cumming and Sinclair (2009) mention an undescribed species from Costa Rica. There are two species recorded for Brazil: *M. barueri* Smith from São Paulo and *M. breviradius* Smith from Santa Catarina (Smith 1962). The remaining Neotropical species were described from the extreme south of South America, in southern Argentina and southern Chile (Collin 1933). Herein, two new species are described from southeastern Brazil, and a key to the Neotropical species is provided.

## Materials and methods

This study is based on the examination of specimens housed at Instituto Nacional de Pesquisas da Amazônia, Manaus, Amazonas, Brazil (**INPA**). Species with long series of representatives will also be deposited in the Museu Nacional do Rio de Janeiro (**MNRJ**), and Museu de Zoologia da Universidade de São Paulo (**MZUSP**). The specimens were collected using Malaise traps placed over small streams.

Dissected structures were macerated in heated 85% lactic acid (Cumming 1992) and examined on excavated slides. Wings were mounted on microslides, terminalia were placed in microvials with glycerin, and these were pinned with their associated specimens. Terminology follows Cumming and Wood (2009).

The holotype label data was cited in full before the description, with original spelling and punctuation. Data from each label was enclosed by quotation marks (“ ”). Information presented within square brackets ([]) is supplementary data not present on the labels.

## Taxonomy

### Metachela

Taxon classificationAnimaliaDipteraEmpididae

Metachela Coquillett, 1903: 253, 263. Type species: Hemerodromia collusor Melander, 1902 (original designation). Melander 1928: 262 (cat.); Collin 1933: 285 (Patagonian fauna); Melander 1947: 260 (cat.); Smith 1962: 261 (Brazilian fauna); Smith 1967: 42 (Neotropical cat.); MacDonald 1989: 513 (Nearctic fauna); Yang et al. 2007: 276 (world cat.); Cumming and Sinclair 2009:667 (undescribed Costa Rican species).

#### Diagnosis.

Face with some pale setae, front tibiae with apical rather trowel-like projection beneath and no spine, crossvein *h* present, cells bm and dm fused (crossvein bm-cu absent), M_1_ and M_2_ with common petiole arising from anterior end of crossvein dm-cu and cell *CuP* present. The Neotropical *Metachela* differs from the typical northern species by lacking a distinct stylus, having the thorax more pointed anteriorly, possessing four to equally spaced vertical setae and lacking spine below the front tibia. Although there are the differences mentioned, we still think that the Neotropical species are congeneric with the northern species.

#### Key to the Neotropical species of *Metachela*

**Table:** 

1	Pterostigma semi-circular, almost closed by a veinlet (Fig. 24). Records: Argentina, Bariloche	***M. circumdata* Collin**
–	Pterostigma absent (Figs 2, 20)	**2**
2	Ground colour of thorax tawny or yellow, never black	**3**
–	Ground colour of thorax black	**8**
3	Scutum with a mid-longitudinal brown stripe (Fig. 12)	**4**
–	Scutum without a mid-longitudinal stripe	**7**
4	Vein R_2+3_ ending on C; fore femur very stout. Records: Argentina, Bariloche	***M. patula* Collin**
–	Vein R_2+3_ fused to vein R_1_ (Figs 2, 9, 20); fore femur not stout	**5**
5	Scutellum yellow. Records: Brazil, Santa Catarina	***M. breviradius* Smith**
–	Scutellum brown (Figs 1, 8, 12)	**6**
6	Male cercus with acute apex (Fig. 5), without spine-like setae (Fig. 3); epandrium with posterodorsal sinus; female tergite 10 bilobate on posterior margin (Fig. 11). Records: Brazil, Minas Gerais and Rio de Janeiro	***M. danitakiyae* sp. n.**
–	Male cercus with truncate apex, and with scattered spine-like setae (Figs 15 and 16); epandrium without posterodorsal sinus; female tergite 10 divided into two sclerotized plates separated by a membranous area (Fig. 22). Records: Brazil, Minas Gerais	***M. spinulosa* sp. n.**
7	Scutum entirely yellow. Abdominal segments 4 and 5 brownish. Records: southern Chile	***M. flavella* Collin**
–	Scutum with a short dark streak below the notopleural setae and slightly dark anterior to the scutellum. Abdomen segments 4 and 5 yellowish. Records: Brazil, São Paulo	***M. barueri* Smith**
8	Head with six vertical setae. Records: southern Chile	***M. hexachaeta* Collin**
–	Head with four vertical setae	**9**
9	Scutum with alternating dark and pale stripes. Mid femora with yellow posteroventral setae at base and an anteroventral row of denticles towards the apex. Records: southern Chile	***M. instabilis* Collin**
–	Scutum entirely black. Mid femora without distinctive setae or denticles. Records: Argentina, Bariloche	***M. inornata* Collin**

### Metachela danitakiyae sp. n.

Taxon classificationAnimaliaDipteraEmpididae

http://zoobank.org/D957CD88-DFBC-4544-A465-1BB95F63082F

Figs 1–7 , 8–11

#### Type-locality.

BRAZIL, Rio de Janeiro: Itatiaia, Parque Nacional de Itatiaia, 22°25'38.6"S–44°37'9.7"W, 1140 m,

#### Type-specimen.

Holotype male, pinned, not dissected: “BRAZIL, RJ [Rio de Janeiro], Itatiaia, Parque Nacional de Itatiaia. Córrego Maromba, abaixo da Cachoeira Véu de Noiva. Malaise trap, 22°25'38.6"S–44°37'9.7"W, 1140 m. 10.i–02.II.2015. D.M. Takiya, A.P.M. Santos & M.F. Monné” (INPA). Paratypes. Same data as holotype (8 males, 2 females, INPA, 5 males, 2 females, MNRJ, 3 males, 4 females, MZUSP). BRAZIL, MG[Minas Gerais], Alto Caparaó, Parque Nacional do Caparaó, Vale Verde. Malaise, 17–20.i.2014. 20°25'09.7"S–41°50'47"W, 1364m. J.L. Nissimian & A.P.M. Santos. (2 males, 1 female, INPA).

#### Diagnosis.

Scutum with a mid-longitudinal brown stripe; vein R_2+3_ fused to R_1_ (Figs 2 and 9); male cercus arched, in dorsal view, acute at extreme apex (Figs 3, 4); epandrium with a dorsoapical sinus (Fig. 5); hypandrium membranous midventrally on basal ¾ and sclerotized apically (Fig. 7); female tergite 10 with bilobate posterior margin (Fig. 11); female sternite 8 elongate, concave on anterior margin, membranous on apical half (Fig. 11).

**Figures 1–7.**
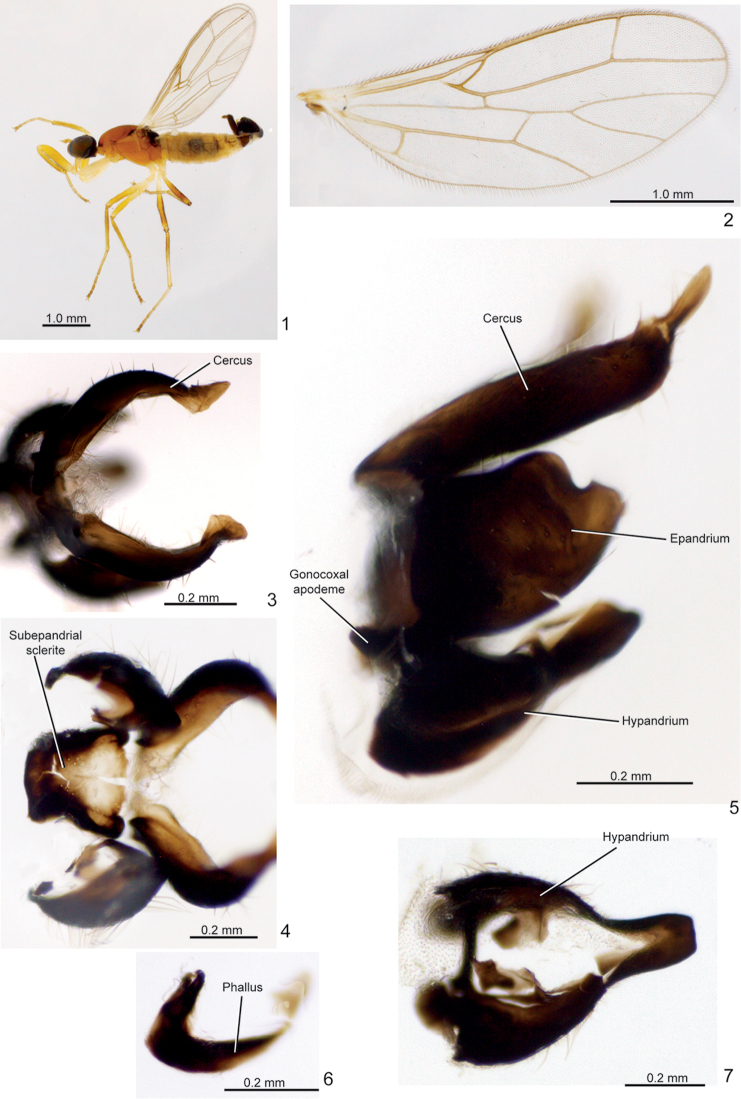
*Metachela danitakiyae* sp. n., **1–2** holotype male **3–7** paratype ♂ **1** Habitus, lateral view **2** Wing **3** Cercus, dorsal view **4** Subepandrial sclerite, epandrium and cercus, anteroventral view **5** Cercus, epandrium and hypandrium, lateral view **6** Phallus, lateral view **7** Hypandrium, ventral view.

***Male*** (Fig. 1). *Head* (Fig. 1): Dark brown to black, setae whitish. Ocellar triangle with two pairs of proclinate bristles, anterior pair stouter. Eyes iridescent black, separated on face. Occiput with scattered fine setae. Mouth parts yellow; proboscis short, slightly curved and with yellow setae. Antenna yellow, with scape and pedicel bearing distinct short ventral setulae; postpedicel nearly 2× as long as wide; stylus very short, ~ 0.1× as long as postpedicel.

*Thorax* (Fig. 1): Elongate, slightly arched dorsally; scutum yellowish except for mid-longitudinal brown stripe, wider posteriorly, and brownish posterolateral spot above wing base; scutum with very small and fine yellow setae except for one notopleural, one postalar, and two pairs of small parallel scutellars; scutellum and mediotergite brown.

*Legs* (Fig. 1): Yellow, except fore tibia with narrow anteroventral brown stripe distally, and hind femur at distal 2/3 and tarsomeres 4–5 brown. Fore coxa as long as distance between fore- and mid coxae, 4× as long as wide, with some dorso-apical pale setae. Fore femur approximately 1.3× as long as fore coxa, 4.5× as long as wide, with anteroventral row of 3–5 spines, anteroventral row of 6–8 denticles, and posteroventral row of 18–20 denticles, with basal ones stouter; anteroventral row of denticles placed on distal half and posteroventral row restricted to distal 0.9, and both rows without apical discontinuity and diverging at apex. Fore tibia approximately 0.8× as long as fore femur; with decumbent short pale bristles dorsally, more densely distributed apically. Mid femur with two anteroventral and 12 posteroventral spines, basal pair stouter. Hind legs slender, with fine setae, except hind tibia with dorsoapical comb of short setae.

*Wings* (Fig. 2): Membranous, veins yellowish; vein R_2+3_ short, fused to vein R_1_; R_4+5_ fork angle around 70°; R_5_ and M_1_ slightly divergent at extreme apex; cell bm+dm ending beyond apex of R_1_, ~1.3× as long as cell br; cup cell closed. Halter whitish yellow.

*Abdomen* (Fig. 1): Tergites and sternites membranous, yellow, except anterior margin of all tergites and sternite 8 brownish; sternite 8 strongly sclerotized, U-shaped posteriorly.

*Male terminalia*: Brown. Cercus arched, in dorsal view (Figs 3, 4) narrower on basal 1/4, expanded apically, extending beyond epandrium apex (Fig. 5); left and right cerci closely approximated anterodorsally (Fig. 3); distinctly setose. Epandrium subrectangular, with a posterodorsal sinus (Fig. 5), with distinct strong setae on outer face. Hypandrium membranous midventrally on basal 3/4, sclerotized and fused posteriorly (Fig. 7); gonocoxal apodeme projecting anteriorly as a small protuberance (Fig. 5). Subepandrial sclerite subrectangular, more sclerotized basally and laterally (Fig. 4). Phallus strongly sclerotized, except less sclerotized apically (Fig. 6), abruptly pointed apically. Ejaculatory apodeme present. Holotype: body length. 3.3 mm; wing length. 2.7 mm.

**Figures 8–11.**
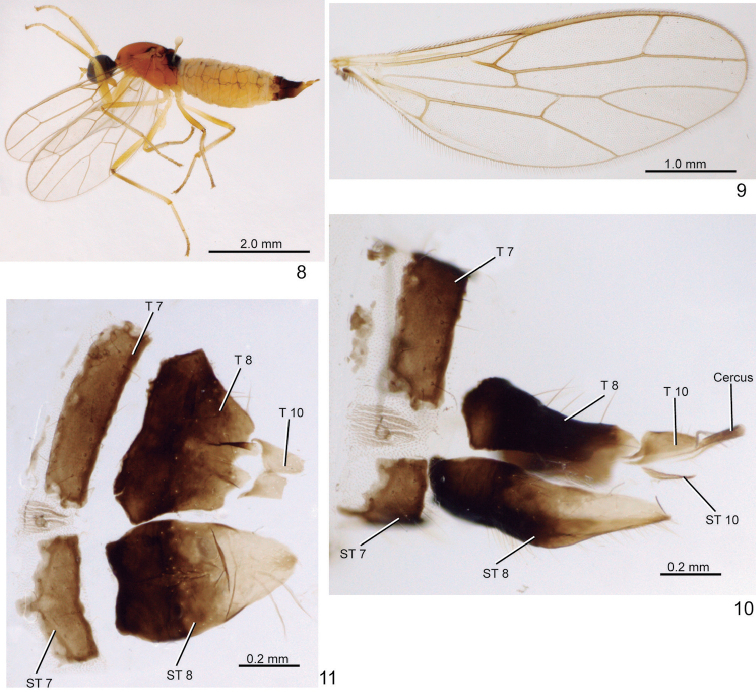
*Metachela danitakiyae* sp. n., paratype female from Itatiaia. **8** Habitus, lateral view **9** Wing **10** Segments 7-11, lateral view **11** Tergites 7, 8, and 10, dorsal view, sternites 7 and 8, ventral view.

***Female*** (Figs 8, 9). Similar to male. Terminalia: Tergite 7 brown, shorter than tergite 8 (Figs 10, 11); tergite 8 brown, subtrapezoidal, with anterior margin slightly concave in dorsal view (Fig. 11); tergite 10 slightly light brown, bilobate on posterior margin (Fig. 11). Cercus brown, apex pale (Fig. 10). Sternite 7 brown, with small median projection on anterior margin (Fig. 11); sternite 8 dark brown on basal 2/3 and with pale apex, elongate, concave on anterior margin (Fig. 11); sternite 10 very narrow, v-shaped.

#### Geographical records.

Brazil (Minas Gerais and Rio de Janeiro states).

#### Etymology.

The specific epithet is a tribute to Daniela Maeda Takiya, friend of the authors and collector of the specimens.

#### Remarks.

*Metachela danitakiyae* sp. n. differs from other species by the elongate male cercus, narrower on the apical 1/4 (usually short in other species, if elongate then with broad apex); epandrium with a posterodorsal sinus (without sinus in other species).

### Metachela spinulosa sp. n.

Taxon classificationAnimaliaDipteraEmpididae

http://zoobank.org/740F1C97-E46A-40DE-8F24-97FE22ECBEF5

Figs 12–18 , 19–23

#### Type-locality.

BRAZIL, Minas Gerais, São Roque de Minas, Parque Nacional Serra da Canastra, Rio Rolador.

#### Type-specimen.

Holotype male, pinned, with abdomen in a microvial. Original label: “BRAZIL, **MG[Minas Gerais**], São Roque de Minas, Parque Nacional Serra da Canastra, Rio Rolador. Malaise, 15–18.xi.2014. J.L.Nissimian, A.L. Oliveira & A.P.M. Santos.” (INPA). Paratypes. Same data as holotype (1 male, 3 females, INPA).

#### Diagnosis.

Scutum with a mid-longitudinal brown stripe; vein R_2+3_ fused to R_1_; male cercus with truncate apex and somewhat scattered spine-like setae; epandrium with an apicoventral acute projection; female tergite 8 with bilobate apex; female tergite 10 divided into two sclerotized plates separated by a membranous area.

**Figures 12–18.**
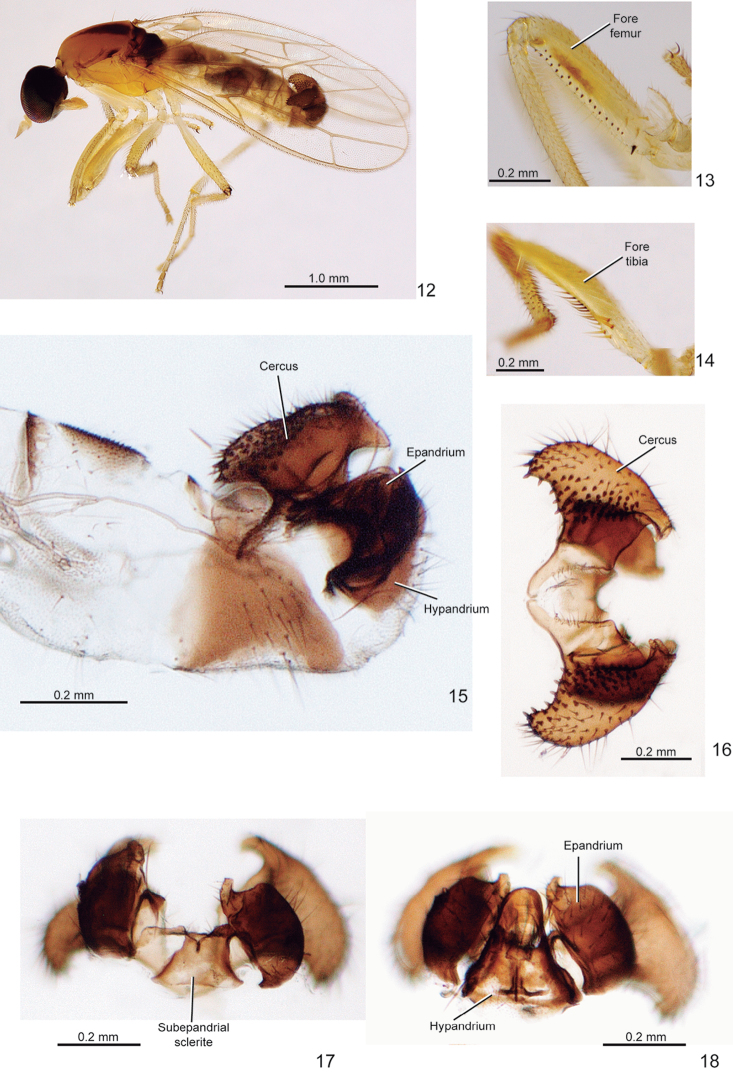
*Metachela spinulosa* sp. n., holotype male. **12** Habitus, dorsolateral view **13** Right fore femur, anteroventral view **14** Right fore tibia, anteroventral view **15** Abdomen from segments 7–11, lateral view **16** Cercus, dorsal view **17** Subepandrial sclerite and epandrium, dorsal view **18** Hypandrium and epandrium, ventral view.

***Male*** (Fig. 12). Holotype: body length: 3.5 mm; wing length: 2.7 mm.

*Head* (Fig. 12): Dark brown to black, setae whitish. Ocellar triangle with two pairs of proclinate bristles, anterior pair stouter. Eyes iridescent black, separated on face. Occiput with scattered fine setae. Mouth parts yellow; proboscis short, right and with yellow setae. Antenna yellow, with scape and pedicel bearing distinct short ventral setulae; postpedicel approximately 2× as long as wide; stylus very short, 0.1× as long as postpedicel.

*Thorax* (Fig. 12): Elongate, slightly arched dorsally; scutum yellowish except for mid-longitudinal brown stripe, darker posteriorly, and brownish posterolateral spot above wing base; scutum with very small and fine yellow setae except for one notopleural, one postalar, and two pairs of small parallel scutellars; scutellum and mediotergite brown;

*Legs* (Fig. 12): Yellow. Fore coxa as long as distance between fore- and mid coxae, 3× as long as wide, with some dorsoapical pale setae. Fore femur (Fig. 13) 1.2× as long as fore coxa, 3.5× as long as wide, with anteroventral row offour spines, anteroventral row of 4 denticles, and posteroventral row of 17 denticles, with basal one stouter; anteroventral row of denticles placed on distal half and posteroventral row restricted to distal 0.9, and both rows without apical discontinuity and diverging at apex. Fore tibia (Fig. 14) ~ 0.8× as long as fore femur; with decumbent short pale setulae dorsally, denser apically. Mid femur with 2 anteroventral and 15 posteroventral spines, basal pair stouter. Hind legs slender with fine setae, except hind tibia with dorsoapical ‘comb’ of short setae.

*Wings* (Figs 12 and similar to 20 of female): Membraous, veins yellowish; vein R_2+3_ short, fused to R_1_; R_4+5_ fork angle around 70°; R_5_ and M_1_ slightly divergent at extreme apex; cell bm+dm ending beyond apex of R_1_, ~ 1.4× as long as cell br. Halter whitish yellow.

*Abdomen* (Fig. 12): Tergites and sternites 1–6 and anterior margin of tergite 7 yellowish, membranous; posterior margin of tergite 7, tergite 8 and sternite 8 brownish; sternite 8 strongly sclerotized, U-shaped with lateral side upward directed posteriorly.

*Male terminalia*: Brown. Cercus wider on basal 1/3, apex truncate in lateral view (Fig. 15), with somewhat scattered spine-like setae (Fig. 16); left and right cerci closely approximated anterodorsally (Fig. 16). Epandrium with a posterodorsal pointed projection apically (Figs 17 and 18) and distinct strong setae on outer face (Fig. 17). Hypandrium membranous medially on basal half, with strong setae (Figs 15 and 18); gonocoxal apodeme projecting anteriorly as a small protuberance. Subepandrial sclerite subrectangular, more sclerotized basally and laterally (Fig. 17). Phallus strongly sclerotized, abruptly acute apically. Ejaculatory apodeme short, trilamellar.

**Figures 19–24.**
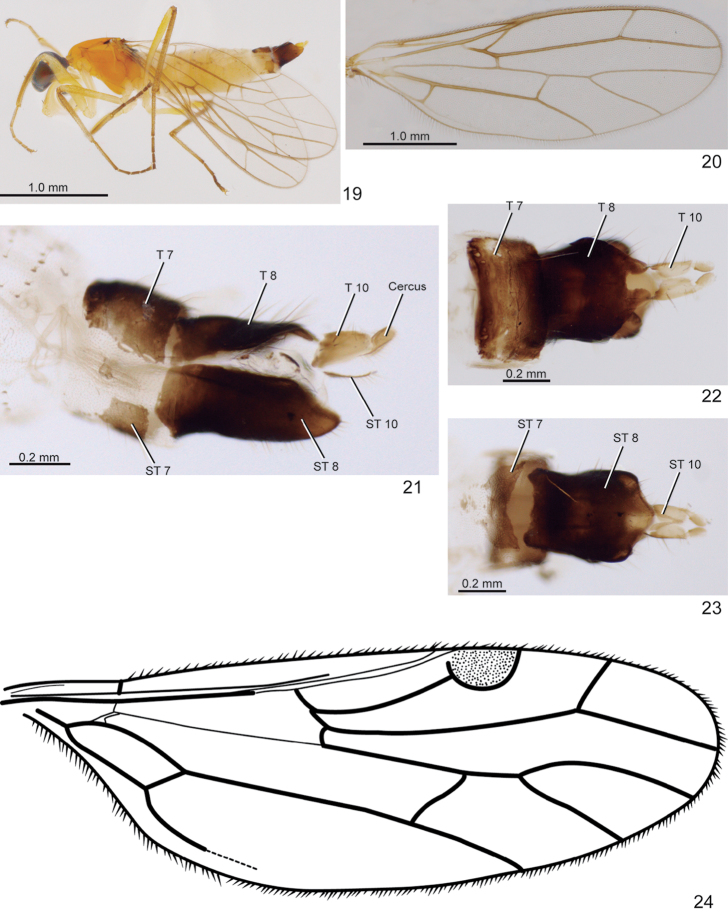
**19–23** *Metachela spinulosa* sp. n., paratype female **19** Habitus, lateral view **20** Wing **21** Segments 6-11, lateral view **22** Tergite 7 until cercus, dorsal view **23** Segments 7-11, ventral view **24** *Metachela circumdata*, wing modified from Collin (1933).

***Female*** (Fig. 19, 20). Similar to male. Tergite 7 brown, rectangular, shorter than tergite 8 (Figs 21 and 22); tergite 8 brown, elongate, bilobate posteriorly (Fig. 22); tergite 10 light brown, divided into two sclerotized plates separated by membranous area medially (Fig. 22). Cercus light brown, apex pale (Figs 21, 22 and 23). Sternite 7 light brown, concave on posterior margin (Fig. 23); sternite 8 brown, subrectangular (Fig. 23); sternite 10 light brown, v-shaped (Fig. 23). Body length: 3.9 mm; wing length: 3.1 mm.

#### Geographical records.

Brazil (Minas Gerais).

#### Etymology.

From the Latin *spinosus* (spine), referring to the spine-like setae on the male cercus.

#### Remarks.

*Metachela spinulosa* sp. n. differs from other species especially by the male cercus with scattered spine-like setae (absent in other species) and epandrium with an apicoventral acute projection (absent in other species).

## Discussion

The Atlantic forest is one of the five most important biodiversity hotspots in the world (Myers et al. 2000). In face of the rapid anthropic changes to this area, it is important that its fauna be studied, including Diptera, before of it is lost. Prior to the current study, there were only two species of *Metachela* described from this biome, and no doubt there are certainly new species still left to be described.

## Supplementary Material

XML Treatment for Metachela

XML Treatment for Metachela danitakiyae

XML Treatment for Metachela spinulosa
